# Commentary: Modulation of ASC-derived extracellular vesicles containing cargo that specifically enhances wound healing

**DOI:** 10.3389/fphar.2026.1818729

**Published:** 2026-05-20

**Authors:** Tae-Ho Kim, Jin-Chul Heo, Jong-Ha Lee, Dong-Sun Lee, Hee-Jeong Choi, Sang-Han Lee

**Affiliations:** 1 Biomedical Research Institute, Kyungpook National University Hospital, Daegu, Republic of Korea; 2 Department of Biomedical Engineering, Keimyung University, Daegu, Republic of Korea; 3 Department of Biomaterials Science and Technology, Jeju National University, Jeju, Republic of Korea; 4 Korean Medicine (KM) Application Center, Korea Institute of Oriental Medicine, Daegu, Republic of Korea; 5 Department of Food Science and Biotechnology, Kyungpook National University, Daegu, Republic of Korea; 6 Food and Bio-Industry Research Institute, and Inner Beauty/Antiaging Center, Kyungpook National University, Daegu, Republic of Korea

**Keywords:** adipose-derived stem cells, extracellular vesicles, potency assay, skin repair, wound healing

## Introduction

Recent advances in extracellular vesicle (EV)-based therapeutics have highlighted their potential as biologically active mediators in tissue repair and regenerative pharmacology. In this context, the study by Gurney et al. (2025) presents a manufacturing-driven approach to generating functionally ‘tuned’ EVs for wound healing applications. Here, we provide a critical and constructive commentary focusing on interpretability, reproducibility, and translational considerations.

The authors report a bioreactor-based strategy to manufacture “tuned” extracellular vesicles (EVs) from human adipose-derived stem cells (ASCs), termed eXo3, and benchmark these EVs against “native” EVs generated from the same ASC lot under traditional culture conditions (Gurney et al., 2025). The core premise that *ex vivo* environmental cues can reshape EV cargo and thereby shift functional potency toward a pro-healing phenotype addresses a practical bottleneck in regenerative pharmacology: how to preserve efficacy and consistency while minimizing phenotypic drift (including senescence-associated changes) in producer cells. The manuscript integrates EV characterization with functional *in vitro* assays in primary human skin-relevant cells, paired *in vivo* wound models, and a translational bridge via topical formulation and a single case observation in a chronic diabetic wound setting. Beyond acute wound closure, these questions also matter for inflammatory skin states where impaired repair and inflammation coexist (e.g., selected inflammatory skin contexts), where modulating inflammatory signaling can influence disease trajectory (Lee CH et al., 2022a; Seo et al., 2024).

## Discussion

### What this work adds

For clarity, we use “ASC-derived EVs” to refer to vesicles isolated from adipose-derived stem cells under defined culture conditions, whereas “native EVs” denote vesicles obtained under conventional culture without tuning. Where applicable, conditioned media-derived exosome preparations are explicitly distinguished as a separate comparator. Terminology is used consistently throughout.

A notable contribution is the manufacturing-centric framing of EV potency. The authors highlight that a controlled hollow-fiber bioreactor environment can be leveraged to tune EV production and cargo packaging, positioning process parameters as actionable levers for improving bioactivity (Gurney et al., 2025). The characterization panel is recognizable and practical, including nanoparticle tracking analysis (ZetaView NTA), transmission electron microscopy (TEM), and surface marker profiling using the MACSPlex Exosome Kit (37 markers), complemented by BCA-based protein quantification. In a field where nomenclature, separation, and characterization rigor remain recurrent review points, a brief statement of alignment with MISEV-style reporting items would further support interpretability and reproducibility (Théry et al., 2018; Welsh et al., 2024).

### Enhancing clarity around comparators and EV source terminology

The parallel preparation of eXo3 and “native” EVs from the same ASC lot is a strength because it helps isolate the effects of manufacturing cues (Gurney et al., 2025). It may help readers if the manuscript briefly reiterates (at a high level) which bioreactor environmental features are modified for eXo3 versus native conditions and how these are controlled across runs.

Clarifying whether the *in vivo* ‘exosome preparation derived from adipocyte-conditioned media’ is identical to the ASC-derived EVs described in Sections 2.1–2.2, or represents a distinct input, would materially improve traceability from manufacturing to outcomes.

This single clarification would make it easier to map production inputs to downstream outcomes, and it would support the manuscript’s emphasis on controlled manufacturing and product definition (Théry et al., 2018; Welsh et al., 2024).

### Potency assays: strong coverage, with opportunities for tighter assay-to-endpoint linkage

The *in vitro* panel is well aligned with key features of skin repair, capturing inflammatory cytokine modulation, migration, proliferation, and matrix output in primary human skin-relevant cells (Gurney et al., 2025). For example, epithelial/keratinocyte migration and proliferation are most directly associated with re-epithelialization and closure kinetics; fibroblast proliferation and procollagen-1 output align more with granulation tissue formation and matrix deposition; and cytokine suppression (e.g., IL-6/IL-8/MCP-1) is linked to inflammatory resolution that can influence both closure and remodeling. Keratinocyte migration and proliferation most directly reflect re-epithelialization and early closure kinetics, whereas fibroblast proliferation and procollagen-1 output map to granulation tissue formation and extracellular matrix deposition that shape repair strength and remodeling. Cytokine modulation (e.g., IL-6/IL-8/MCP-1) provides a complementary indicator of inflammatory resolution, which can influence both the rate of closure and the quality of tissue maturation. Stating this assay-to-endpoint mapping upfront would help readers interpret ‘potency’ as linked to defined healing phases rather than as a broad assay list.

### *In vivo* wound models: strong design elements and suggestions for endpoint specificity

The excisional wound section includes several elements reviewers commonly value: standardized dorsal wounds, within-animal PBS vehicle controls, randomization across wound positions to reduce positional bias, and blinded assessment (Gurney et al., 2025). The use of daily wound measurement and photography for up to 30 days supports kinetic analysis and durability assessment.

To strengthen biological interpretability, it would be useful to clarify whether the primary focus is surface closure kinetics, repair quality, or both. For closure kinetics, this could be stated explicitly as serial wound area reduction over time (e.g., time to 50% and complete closure). For repair quality, a minimal set of end-of-study readouts—such as re-epithelialization thickness/continuity, collagen organization (Masson’s trichrome or picrosirius red), and a semi-quantitative inflammatory infiltrate score—would help distinguish faster closure from improved tissue maturation.

### Context and positioning within the broader ASC-EV wound-healing landscape

A growing preclinical literature supports the concept that adipose-derived stem cell exosomes can accelerate wound healing and improve vascularization, collagen deposition, and re-epithelialization, including in diabetic wound settings (Wei et al., 2024). Given this background, the present work’s emphasis on manufacturing control and potency consistency is timely and potentially differentiating.

At the same time, the manuscript’s discussion of senescence and SASP-like drift is an important framing choice; recent literature has explicitly highlighted “senescent drift” risk as a quality and efficacy issue for MSC/ASC secretome-based therapeutics in skin wound repair (Smirnova et al., 2023). In that sense, additional short clarifications about how senescence is operationally monitored/avoided in the production pipeline would likely be received positively.

### Translational framing: topical formulation and case observation

The topical translation is of interest, particularly because eXo3 is incorporated into a hyaluronic acid-based gel for OTC personal care use, whereas PBS dilution is used for *in vitro* and animal assays (Gurney et al., 2025). In the single diabetic wound case observation, we recommend stating explicitly that this is hypothesis-generating and intended to illustrate translational feasibility rather than demonstrate efficacy. Where applicable, a brief note indicating that informed consent was obtained and whether standard-of-care measures or other co-interventions were continued would help readers interpret the case observation appropriately. One concise limitations sentence (e.g., no randomization/blinding, possible co-interventions/standard care changes, and natural fluctuation in chronic wounds) would preserve credibility while keeping the real-world signal visible.

## Conclusion

Overall, this study provides a timely and practically relevant contribution by connecting controlled bioreactor production of ASC-derived EVs with a practical potency and wound-healing evaluation package (Gurney et al., 2025). The study’s impact could be further strengthened by minor reporting clarifications (particularly around comparator definitions and source terminology), along with a tighter linkage between *in vitro* assays and *in vivo* endpoints and clearer specification of “healing” endpoints beyond closure. We offer a minimal checklist (Table 1) aligned to the methods and models used here as a constructive tool to support reproducibility and facilitate evaluation of EV-based dermatologic repair claims. More broadly, establishing a rigorous potency-and-endpoint framework may help inform future, carefully controlled exploration of EV-based strategies in chronic inflammatory skin settings where impaired repair and immune dysregulation coexist, including atopic dermatitis–associated barrier dysfunction contexts (Lee et al., 2022a; Lee et al., 2022b).

**TABLE 1:** Minimal checklist for EV definition, dosing, controls, and endpoints in EV-based wound repair.

Category	Minimal items to report/Verify	Why it matters
EV definition and comparators	Use consistent ‘EV’ terminology; map all comparator labels to the source and isolation pipeline	Enables input-to-outcome traceability
Producer cells and process	Report ASC donor/lot and passage range; summarize tuned process features and number of independent batches	Addresses major sources of EV variability
Isolation and storage	State isolation method/critical parameters; report storage conditions and freeze–thaw history	Purity and activity can shift with handling
Core EV characterization	Provide NTA (size + concentration), TEM, and marker profiling; include a negative/contamination indicator when feasible	Supports identity, purity, and comparability
Dose and controls	Report dose as particles or µg protein (with quantification); include vehicle and (for topical) matrix-only controls; specify positive controls if used	Separates EV effects from vehicle/matrix effects
Potency readouts	Define primary human skin-relevant assays and key readouts/timepoints (inflammation, migration, proliferation, matrix output)	Clarifies what ‘potency’ means biologically
Assay-to-endpoint linkage	Briefly map *in vitro* readouts to *in vivo* endpoints (re-epithelialization, granulation/remodeling)	Strengthens the potency-to-healing rationale
*In vivo* models and endpoints	Report model details, randomization/blinding, treatment schedule/route; state whether outcomes emphasize closure kinetics, repair quality, or both (list core quality endpoints if assessed)	Defines ‘healing’ and improves interpretability
Measurement and statistics	Describe planimetry/imaging standardization; specify unit of analysis (wound vs. animal) and handling of clustered/repeated measures	Improves statistical validity
Translational claims	For human observations, label as hypothesis-generating; report standard care/co-interventions and quantification approach	Limits over-claiming and preserves credibility
